# LncRNA MSC-AS1 Promotes Colorectal Cancer Progression by Regulating miR-325/TRIM14 Axis

**DOI:** 10.1155/2021/9954214

**Published:** 2021-05-11

**Authors:** Changhong He, Xia Wang, Meichun Du, Yanjun Dong

**Affiliations:** ^1^Department of Clinical Laboratory, Yantaishan Hospital, Yantai 264000, China; ^2^Blood Purification Centre, East Hospital, Qingdao Municipal Hospital, Qingdao 266071, China; ^3^PICC Clinic, Qingdao Central Hospital Affiliated to Qingdao University, Qingdao 266042, China; ^4^Department of Clinical Laboratory, People's Hospital of Rizhao, Rizhao 276800, China

## Abstract

**Background:**

LncRNA MSC-AS1 has been reported to be a tumor promoter in hepatocellular carcinoma. However, the function of MSC-AS1 in colorectal cancer (CRC) has not been elucidated. It is designed to study the expression level of MSC-AS1 and investigate its biological effect on the progression of CRC.

**Methods:**

The expression patterns of MSC-AS1, miR-325, and TRIM14 were explored by RT-qPCR in CRC tissues and cells. The protein expression of TRIM14 was tested by Western blot assay. The association between MSC-AS1 expression and clinicopathological data was analyzed by chi-squared test. CCK-8 assay, colony formation, and Transwell assay were used to investigate the effect of MSC-AS1 on cell growth, invasion, and migration in CRC cells. The correlations among MSC-AS1, miR-325, and TRIM14 were analyzed by Pearson's correlation coefficient analysis.

**Results:**

We found that MSC-AS1 and TRIM14 were upregulated in CRC tissues, while miR-325 was downregulated in CRC tissues. Functional experiments demonstrated that MSC-AS1 knockdown inhibited cell proliferation, migration, and invasion abilities in CRC cells. Additionally, miR-325 was proved to be a target miRNA of MSC-AS1, and TRIM14 might be a downstream gene of miR-325. Besides that, MSC-AS1 counteracted the inhibitory effect of miR-325 on the cell progression and TRIM14 expression.

**Conclusion:**

Our results indicated that MSC-AS1 facilitated CRC progression by sponging miR-325 to upregulate TRIM14 expression. We suggested that MSC-AS1 might be a potential lncRNA-target for CRC therapy.

## 1. Introduction

Colorectal cancer (CRC) is one of the most common gastrointestinal malignancies, which seriously threatens human health. The incidence of CRC has been on the rise worldwide. In 2020, about 1.9 million patients were diagnosed with CRC worldwide, and more than 935,000 patients died directly or indirectly from CRC [1]. A large number of studies have shown that dietary habits (alcohol consumption, red meat, processed meat, and refined grains, etc.), lifestyle (low physical labor, smoking, etc.), obesity, diabetes, and genetic factors are the most important causes of CRC [2, 3]. The treatment of CRC is generally based on surgery, followed by radiotherapy, chemotherapy, or other treatments [4]. Although the survival rate of CRC has been improved in recent years, the economic burden of CRC ranks first among malignancies. In recent years, molecular targeted therapy has become a hot spot in tumor therapy.

These non-protein-coding RNAs are called noncoding RNAs, including long noncoding RNAs (lncRNAs) with a length of more than 200 nucleotides. Although the mode and regulatory mechanism of lncRNAs in tumors have not been thoroughly studied, lncRNAs are verified to play a vital role in the occurrence and development of human cancers [5]. At present, it has been found that related lncRNAs are involved in the occurrence, metastasis and invasion, early diagnosis, prognosis evaluation, and radiotherapy and chemotherapy efficacy of CRC [6, 7]. LncNEAT1 was reported to promote CRC progression by suppressing miR-486-5p expression and regulating NR4A1/Wnt/*β*-catenin pathway [8]. Lnc-HSD17B11-1:1 acted as a tumor enhancing factor by regulating miR-338-3p and MACC1 in CRC [9]. In addition, lncGNAT1-1 acted as a tumor inhibitor in CRC by modulating RKIP-NF-kappaB-Snail pathway [10]. LncRNA MSC antisense RNA 1 (MSC-AS1) has been reported to accelerate osteogenic differentiation by regulating miR-140e-5p and BMP2 [11]. Moreover, MSC-AS1 promoted tumor progression in kidney renal clear cell carcinoma [12]. Nevertheless, the role of MSC-AS1 in CRC remains unclear.

Plentiful reports have confirmed that lncRNAs act as miRNA sponges to upregulate downstream genes to affect tumor progression. For example, lncRNA UCA1 was found to promote CRC cell proliferation and 5-FU resistance by suppressing miR-204-5p [13]. KCNQ1OT1 was reported to facilitate CRC cell progression by inhibiting miR216b-5p to increase ZNF146 expression [14]. Zhang et al. found that LINC00152 was downregulated in CRC and induced cell apoptosis in CRC cells by regulating miR-376c-3p [15]. miR-325 was found to inhibit cell growth and metastasis by targeting MT3 in bladder cancer [16]. In the current work, bioinformatics analysis confirmed that miR-325 might be a target miRNA of MSC-AS1.

In this article, we explored the expression of MSC-AS1 in CRC tissues. In the meantime, the function of MSC-AS1 knockdown on cell proliferation, migration, and invasion was also detected. Most importantly, we demonstrated that MSC-AS1 influenced the tumor progression by regulating miR-325/TRIM14 axis in CRC.

## 2. Materials and Methods

### 2.1. Clinical Specimens

46 tissues from patients with CRC were obtained from Yantaishan Hospital. The CRC tissues and paracancerous tissues were surgically collected and stored in liquid nitrogen. All patients did not receive any preoperative treatment (radiotherapy or chemotherapy). Before surgery, the purpose and significance of this study were introduced to all patients or their family members; and we have obtained the informed consents signed by all patients or their family members. This study was approved by the Ethics Committee of Yantaishan Hospital.

### 2.2. Cell Culture and Cell Transfection

CRC cell lines HT29, SW620, HCT116, and SW480 and normal human epithelial cells NCM460 were obtained from TongPai (Shanghai) Biotechnology Co., LTD (Shanghai, China). The frozen CRC cells were placed in a 37°C water bath until they were completely dissolved. Cells were resuspended with DMEM containing 10% PBS. Then, CRC cells were cultured in an incubator at 37°C and 5% CO_2_. Cell passage was carried out when the cell confluence reached 70–80%.

CRC cells (2×10^5^ cells/well) in logarithmic growth phase were cultured into 6-well plate. MSC-AS1 siRNA, negative control (si-NC), pcDNA3.1-MSC-AS1 (MSC-AS1 vector), miR-325 mimic, and NC mimic were transfected into cells by Lipofectamine 2000. After culturing at 37°C and 5% CO_2_ for 48 h, follow-up experiments were carried out.

### 2.3. Quantitative Real-Time Polymerase Chain Reaction (qRT-PCR)

TRIzol reagent was performed to extract total RNA from CRC tissues and cells. Then, the concentration of RNA was determined by ultraviolet spectrophotometer. According to the instructions of PrimeScript RT reagent kit, the cDNA was synthesized by reverse transcription. RT-qPCR was performed according to the instructions of SYBR Premix Ex Taq^TM^. The ABI software was used to analyze and process the data to obtain the Ct value. Finally, 2^-△△Ct^ method was used to calculate the relative expression of target gene.

### 2.4. Dual-Luciferase Reporter Assay

StarBase (http://starbase.sysu.edu.cn/) was performed to seek the potential binding miRNAs of MSC-AS1. TargetScan (http://www.targetscan.org/vert_72/) was used to explore the potential downstream genes of miR-325. The mutant and wild sequence fragments of MSC-AS1 and TRIM14 were cloned and combined with the Promega vector. MSC-AS1-Mut and MSC-AS1-Wt and TRIM14-Mut and TRIM14-Wt were transfected into cells with miR-325 mimic or NC mimic, respectively. After transfection for 48 h, the relative luciferase activity of target gene was detected by dual-luciferase reporter assay kit.

### 2.5. MTT Assay

The cells in logarithmic phase were resuspended. 100 *μ*l cell suspension (1 × 10^4^ cells/ml) was inoculated in 96-well plate at 37°C and 5% CO_2_. After incubation for 24, 48, 72, and 96 h, the plates were added with 20 *μ*l MTT solution (5 mg/ml, Sigma) and incubated for another 4 h. Then, 200 *μ*l DMSO was added and shook on the shaker for 10 min. The absorbance value was measured by using a microplate analyzer at 490 nm. According to the experimental data, the growth curve was plotted.

### 2.6. Transwell Assay

For cell migration, after transfection for 48 h, cells were digested with trypsin and resuspended with 400 *μ*l serum-free medium. Cell suspension (1 × 10^5^ cells/ml) was seeded in a 24-well plate. Cells in each group were added to the upper chamber of Transwell chamber, and 600 *μ*L DMEM with 10% FBS was added to the lower chamber of Transwell chamber. Cells were cultured in a constant temperature incubator containing 5% CO_2_ at 37°C for 24 h. Then, the chamber was fixed with 4% formaldehyde for 15 min and stained with crystal violet for 20 min. The migratory cells were observed and photographed under a light microscope. 5 high-power visual fields were randomly selected to count the number of migratory cells.

For cell invasion, 50 *μ*l Matrigel blue was diluted and spread in the upper chamber of Transwell chamber. The remaining steps were the same as those in the cell migration experiment. The invaded cells were observed under a light microscope and photographed. 5 high-power fields were randomly selected to count the number of invaded cells.

### 2.7. Western Blot Assay

Total protein was extracted from CRC cells by TRIzol. The protein samples (30 *μ*g) were boiled at 100°C for 5 min. After SDS-PAGE gel electrophoresis, the protein samples were transferred to the PVDF membranes. After blocking with 5% skim milk for 2 hours, the membranes were incubated with antibodies overnight at 4°C. After incubation with secondary antibodies for another 1 h, the protein signal of CLCA4 was detected by ECL reagent.

### 2.8. Statistical Analysis

All data were expressed as mean ± SD and analyzed by using SPSS 22.0 and GraphPad Prism 6.0. The differences between the two groups were detected by Student's *t*-test. The differences among multiple groups were detected by one-way ANOVA. The correlations between MSC-AS, miR-325, and TRIM14 were detected by Pearson's correlation analysis. *p* < 0.05 was considered statistically significant.

## 3. Results

### 3.1. MSC-AS1 Overexpression Was Discovered in CRC Tissues and Cells

**Figure 1:**
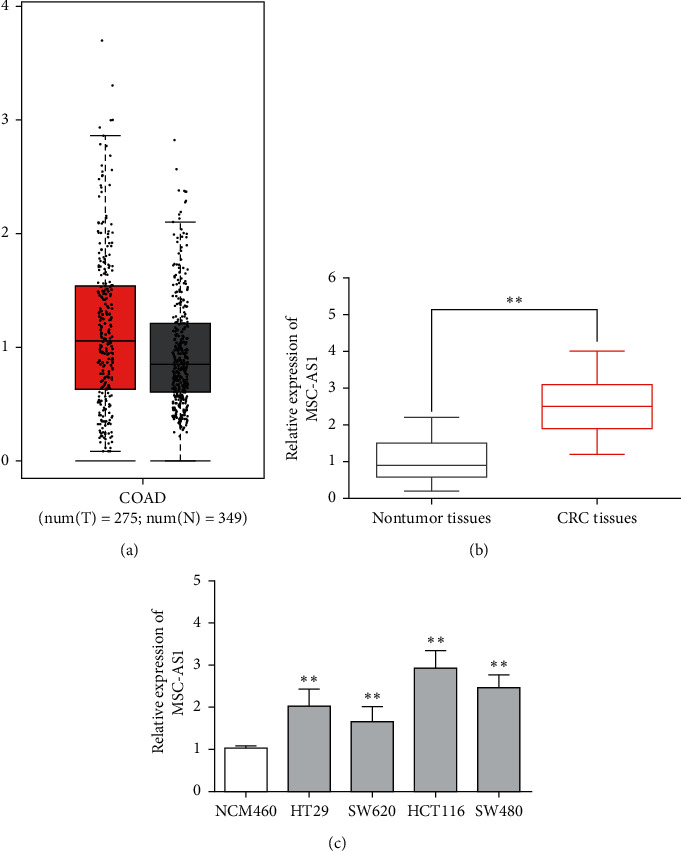
MSC-AS1 expression in CRC tissues and cells. (a) The expression level of MSC-AS1 in GEPIA database. (b) The expression of MSC-AS1 in 46 CRC tissues from Yantaishan Hospital. (c) The expression of MSC-AS1 in CRC cells (HT29, SW620, HCT116, and SW480). ^*∗∗*^*p* < 0.01.

First, GEPIA database showed that MSC-AS1 was upregulated in colon adenocarcinoma (COAD) (Figure 1(a)). Next, RT-qPCR assay was used to detect the expression pattern of MSC-AS1 in 46 CRC tissues from Yantaishan Hospital. It is noted that there was an upward trend of MSC-AS in CRC tissues compared with control tissues (Figure 1(b)). Next, we measured the expression level of MSC-AS1 in CRC cells (HT29, SW620, HCT116, and SW480) and normal human epithelial cells NCM460. As expected, the expression of MSC-AS1 was obviously higher in CRC cells than in NCM460 cells (Figure 1(c)). Furthermore, MSC-AS1 was closely associated with lymph node metastasis (*p*=0.014) and TNM stage (*p*=0.040) (Table 1). Our data indicated that MSC-AS1 was involved in the progression of CRC and might be a potential diagnosis target in patient with CRC.

### 3.2. MSC-AS1 Might Be a Carcinogen in CRC Cells

**Figure 2:**
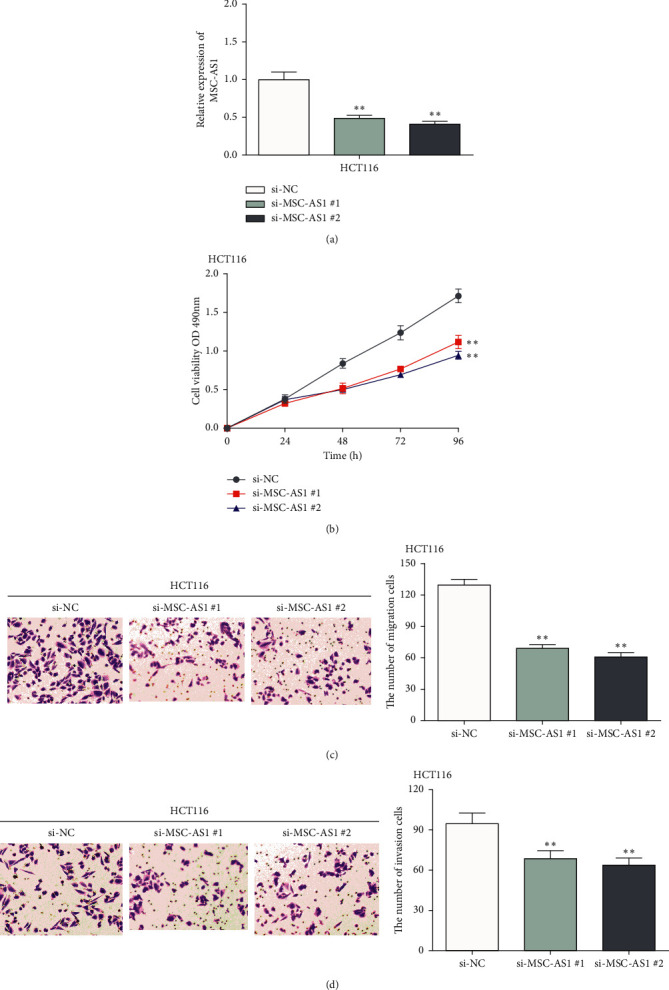
MSC-AS1 might be a carcinogen in CRC cells. (a) The expression of MSC-AS1 was reduced by MSC-AS1 siRNAs. (b) MSC-AS1 knockdown obviously suppressed cell proliferation in HCT116 cells. ((c) and (d)) MSC-AS1 knockdown inhibited cell migration and invasion abilities in HCT116 cells. ^*∗∗*^*p* < 0.01.

In order to investigate the function of MSC-AS1 in CRC, MSC-AS1 siRNA was transfected into HCT116 cells (Figure 2(a)). Then, CCK-8 assay and Transwell assay were performed. CCK-8 results displayed that MSC-AS1 downregulation weakened cell proliferative ability in HCT116 cells (Figure 2(b)). Moreover, Transwell assay displayed that cell migration was obviously inhibited by MSC-AS1 knockdown in HCT116 cells (Figure 2(c)). Similarly, cell invasion ability of CRC cells was blocked by MSC-AS1 knockout (Figure 2(d)). Altogether, our results indicated that MSC-AS1 silencing blocked cell progression in CRC cells.

### 3.3. MSC-AS1 Acted as a Sponge of miR-325 in CRC

**Figure 3:**
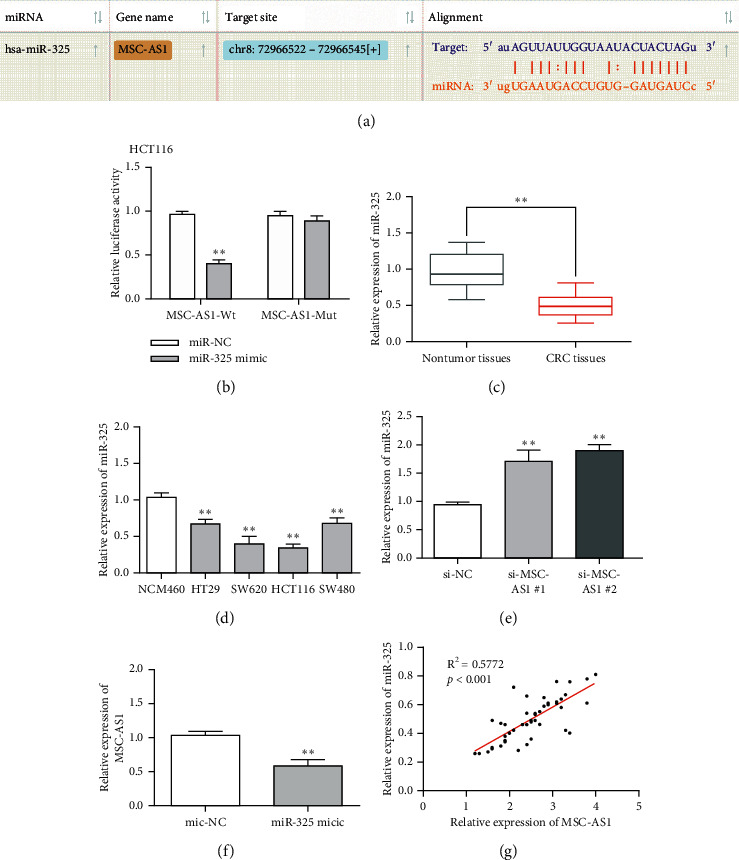
MSC-AS1 acted as a sponge of miR-325 in CRC cells. (a) StarBase showed that there were special binding sites between MSC-AS1 and miR-325. (b) The relative luciferase activity of MSC-AS1-Wt and MSC-AS1-Mut. (c) The expression of miR-325 in CRC tissues. (d) The expression of miR-325 in CRC cells. (e) The expression of miR-325 was increased by MSC-AS1 knockdown. (f) The expression of MSC-AS1 was reduced by miR-325 mimic. (g) Correlation analysis between MSC-AS1 and miR-325 expression in 46 CRC tissues. ^*∗∗*^*p* < 0.01.

In our study, StarBase database was performed to seek the potential miRNAs of MSC-AS1. As shown in Figure 3(a), there were special binding sites between MSC-AS1 and miR-325. Dual-luciferase reporter assay and RT-qPCR assay were performed. We found that the luciferase activity of MSC-AS1-WT was obviously reduced when cells were transfected with miR-325 mimic. However, the luciferase activity of MSC-AS1-MUT was not significantly changed in mimic cells (Figure 3(b)). Next, the expression level of miR-325 in CRC tissues and cells was detected. Results indicated that miR-325 was significantly downregulated in CRC tissues and cells compared with normal groups (Figures 3(c) and 3(d)). Additionally, the expression of miR-325 was increased in HCT116 cells with MSC-AS1 knockdown (Figure 3(e)). Nevertheless, RT-qPCR results indicated that the expression of MSC-AS1 was reduced in CRC cells with miR-325 mimic (Figure 3(f)). Besides, Pearson's correlation analysis was used to detect the relationship between MSC-AS and miR-325 in CRC tissues. The results showed that MSC-AS1 expression was inversely related with miR-325 expression in CRC (Figure 3(g)). In sum, our findings suggested that MSC-AS1 might be a sponge of miR-325.

### 3.4. TRIM14 Might Be a Target Gene of miR-325

**Figure 4:**
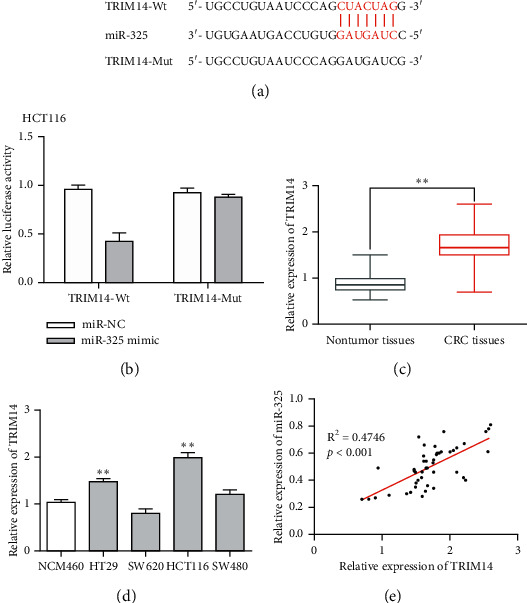
TRIM14 might be a target gene of miR-325. (a) There were special binding sites between miR-325 and TRIM14. (b) The luciferase activity of TRIM14-Wt and TRIM14-Mut. (c) The expression of TRIM14 in CRC tissues. (d) The expression of TRIM14 in CRC cells. (e) Correlation analysis between TRIM14 and miR-325 expression in 46 CRC tissues. ^*∗∗*^*p* < 0.01.

Subsequently, we seek out downstream target gene of miR-325. The StarBase software showed that TRIM14 might be a potential target gene of miR-325 (Figure 4(a)). Dual-luciferase reporter results displayed that miR-325 mimic led to decrease in TRIM14-Wt but not TRIM14-Mut (Figure 4(b)). Then, the mRNA expression of TRIM14 in CRC tissues and cells was detected. We found that TRIM14 was obviously upregulated in CRC tissues compared with nontumor tissues (Figure 4(c)). Furthermore, the expression of TRIM14 was reduced in HT29 and HCT116 cells (Figure 4(d)). Furthermore, there was a negative correlation between miR-325 and TRIM14 in CRC tissues (Figure 4(e)). Our results confirmed that TRIM14 might be a target gene of miR-325.

### 3.5. MSC-AS1 Regulated CRC Progression by Inhibiting miR-325 Expression

**Figure 5:**
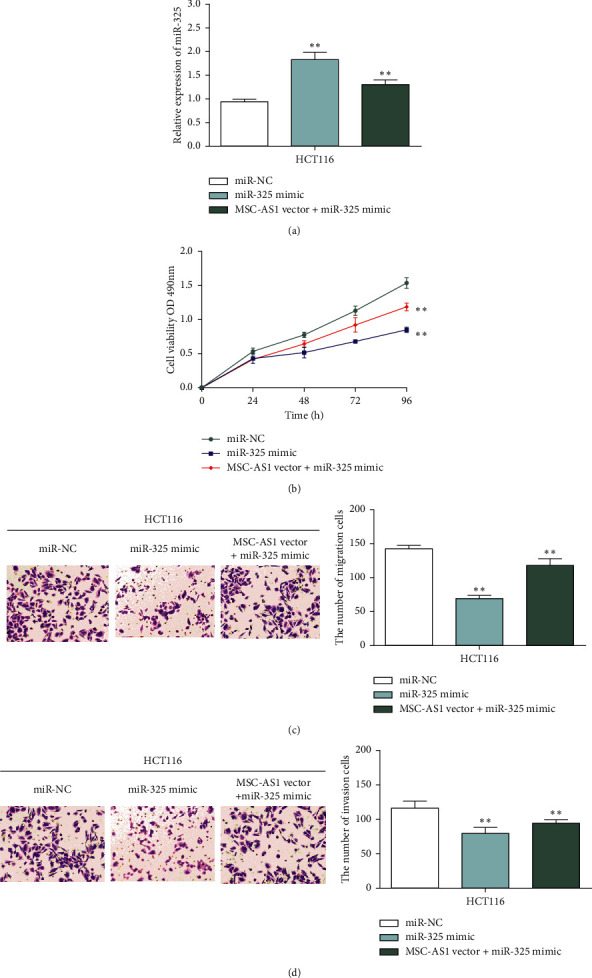
MSC-AS1 regulated CRC progression by inhibiting miR-325 expression. (a) The expression of miR-325 in HCT116 cells transfected with miR-325 mimic or MSC-AS1 vector. (b) miR-325 mimic suppressed cell proliferation, while MMSC-AS1 impaired the inhibitory effect of miR-325. (c, d) miR-325 mimic suppressed cell migration and invasion, while MMSC-AS1 impaired the inhibitory effect of miR-325. ^*∗∗*^*p* < 0.01.

To investigate the mechanism of MSC-AS1/miR-325, MSC-AS1 vector was transfected into CRC cells with miR-325 mimic. RT-qPCR results displayed that the expression of miR-325 was significantly increased in cells when they are transfected with miR-325 mimic, while it declined when mimic cells were transfected with MSC-AS1 vector (Figure 5(a)). Next, CCK-8 assay and Transwell assay were used to measure the function of MSC-AS1/miR-325 in CRC cells. We found that cell proliferative ability was dramatically declined in cells with miR-325 mimic. However, MSC-AS1 overexpression weakened the inhibitory effect of miR-325 mimic on cell proliferation (Figure 5(b)). Furthermore, Transwell results indicated that miR-325 mimic reduced cell migration capability in HCT116 and SW480 cells, while MSC-AS1 transfection reversed the effect of miR-325 (Figure 5(c)). Likewise, MSC-AS1 upregulation destroyed the inhibitory effect of miR-325 mimic on cell invasion ability (Figure 5(d)). Therefore, our data suggested that MSC-AS1 regulated CRC progression by restraining miR-325 expression.

### 3.6. MSC-AS1 Accelerated TRIM14 Expression by Sponging miR-325 in CRC Cells

**Figure 6:**
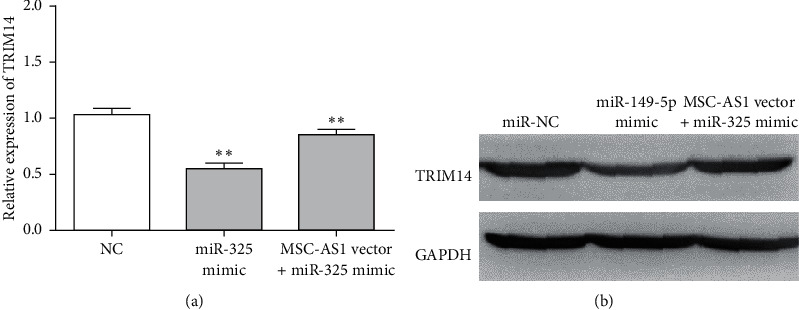
MSC-AS1 accelerated TRIM14 expression by sponging miR-325 in CRC cells. (a) miR-325 mimic reduced the mRNA expression of TRIM14, while MMSC-AS1 impaired the inhibitory effect of miR-325. (b) miR-325 mimic reduced the protein expression of TRIM14, while MMSC-AS1 impaired the inhibitory effect of miR-325. ^*∗∗*^*p* < 0.01.

Next, we explored how MSC-AS1 regulated the TRIM14 expression by targeting miR-325. As shown in Figure 6(a), the mRNA expression of TRIM14 was significantly reduced by miR-325 mimic, while the suppression effect of miR-325 mimic on TRIM14 expression was counteracted by MSC-AS1 vector. In addition, MSC-AS1 vector impaired the inhibitory effect of miR-325 on the protein expression of TRIM14 (Figure 6(b)). Therefore, our data suggested that MSC-AS1 regulated TRIM14 expression by restraining miR-325 expression.

## 4. Discussion

Recently, the mortality and incidence of CRC have been on the rise worldwide, posing a serious threat to human health. Due to the lack of effective CRC prevention and low rate of early diagnosis, most patients are already in the mid and late stages when they are diagnosed. The IARC estimates that, by 2025, there will be more than 20 million new cases of CRC worldwide. In order to find out the effective treatment, it is urgent to study the mechanism of CRC by using molecular biology technology.

At present, the role of lncRNAs in tumor progression has become a research hotspot. Studies have found abnormal expression of lncRNAs in various human cancers, which play a key role in tumor development, metastasis, early diagnosis, prognosis evaluation, radiotherapy and chemotherapy efficacy, etc. Alaiyan et al. reported that CCAT1 was gradually upregulated during the progression of CRC and was associated with tumor metastasis [17]. HAGLR was proved to promote cell growth, migration, and invasion viabilities and inhibit cell apoptosis in CRC [18]. LncRNA MSC-AS1 is a new lncRNA that has been discovered in recent years, which is first found to be overexpressed in hepatocellular carcinoma tissues [19]. Cao et al. confirmed that MSC-AS1 played a carcinogenic lncRNA role in hepatocellular carcinoma by increasing PGK1 expression [20]. As we have mentioned, MSC-AS1 expression was increased in CRC; and we discovered that depletion of MSC-AS1 suppressed tumor progression in CRC. Therefore, our results indicated that MSC-AS1 might be carcinogenic lncRNA in CRC.

Accumulating studies have manifested that lncRNAs can act as ceRNAs to sponge miRNAs and thus upregulate target genes. MSC-AS1 was found to regulate the tumor progression by inhibiting miR-124 expression to increase CDK6 expression in osteosarcoma [21]. Moreover, MSC-AS1 acted as a role of carcinogenic factor in glioma by sponging miR-373-3p and upregulating CPEB4 [22]. Li et al. reported that the expression of miR-325 was reduced in CRC [23]. Similarly, the low expression of miR-325 in CRC was discovered in our study. Additionally, we confirmed that MSC-AS1 might act as a sponge of miR-325 in CRC. Functionally, MSC-AS1 promoted cell proliferation, invasion, and migration in CRC cells by sponging miR-325.

Tripartite motif containing 14 (TRIM14) is a member of the TRIM family. Studies have found that TRIM14 was involved in a variety of biological functions, including cell proliferation, cell apoptosis, inflammatory response, cell metastasis, and immune response [24–26]. TRIM14 was confirmed to facilitate cell proliferation, migration, and invasion and block cell apoptosis in CRC cells [27, 28]. Therefore, TRIM14 was confirmed to play a role as an oncogene in CRC. In the current work, we confirmed that TRIM14 might be a downstream target gene of miR-325. Most importantly, our findings demonstrated that MSC-AS1 regulated TRIM14 expression by restraining miR-325 expression.

Above all, we demonstrated that MSC-AS1 promoted tumor progression by sponging miR-325 to increase TRIM14 expression in CRC. Our results provided a basis for further understanding of CRC progression and supported the potential role of MSC-AS1 as a target for CRC treatment.

## Figures and Tables

**Table 1:** Correlation between the expression level of MSC-AS1 and clinical characteristics of CRC patients (*n* = 46).

Clinical characteristics	Number of cases *n* = 46	MSC-AS1 expression		*p* value
		Low (*n* = 22)	High (*n* = 24)	
*Age (years)*				0.619
≤60	12	5	7	
>60	34	17	17	
*Gender*				0.351
Male	26	14	12	
Female	20	8	12	
*Tumor size*				0.382
≤5 cm	24	10	14	
>5 cm	22	12	10	
*Location*				0.229
Proximal	11	7	4	
Distal	35	15	20	
*TNM stage*				0.040*∗*
I-II	18	12	6	
III-IV	28	10	18	
*Lymph node metastasis*				0.014*∗*
Absent	27	17	10	
Present	19	5	14	

^*∗*^ *p* < 0.05: the difference is significant.

## Data Availability

The data used to support the findings of this study are available from the corresponding author upon request.

## References

[1] Sung H., Ferlay J., Siegel R. L (2021). Global cancer statistics 2020: GLOBOCAN estimates of incidence and mortality worldwide for 36 cancers in 185 countries. *CA: A Cancer Journal for Clinicians*.

[2] Breau G., Ellis U. (2020). Risk factors associated with young-onset colorectal adenomas and cancer: a systematic review and meta-analysis of observational research. *Cancer Control : Journal of the Moffitt Cancer Center*.

[3] Leong K., Hartley J., Karandikar S. (2017). Association of coloproctology of great britain & Ireland (ACPGBI): guidelines for the management of cancer of the colon, rectum and anus (2017) - follow up, lifestyle and survivorship. *Colorectal Disease*.

[4] Carvalho C., Glynne-Jones R. (2017). Challenges behind proving efficacy of adjuvant chemotherapy after preoperative chemoradiation for rectal cancer. *The Lancet Oncology*.

[5] Nandwani A., Rathore S., Datta M. (2021). LncRNAs in cancer: regulatory and therapeutic implications. *Cancer Letters*.

[6] Bermudez M., Aguilar-Medina M., Lizarraga-Verdugo E. (2019). LncRNAs as regulators of autophagy and drug resistance in colorectal cancer. *Frontiers in Oncology*.

[7] Dastmalchi N., Safaralizadeh R., Nargesi M. M. (2020). LncRNAs: potential novel prognostic and diagnostic biomarkers in colorectal cancer. *Current Medicinal Chemistry*.

[8] Liu Z., Gu Y., Cheng X. (2020). Upregulation Lnc-NEAT1 contributes to colorectal cancer progression through Sponging miR-486-5p and Activating NR4A1/Wnt/beta-Catenin pathway. *Cancer Biomarkers*.

[9] Zhang W., Wang B., Wang Q. (2020). Lnc-HSD17B11-1:1 functions as a competing endogenous RNA to promote colorectal cancer progression by sponging miR-338-3p to upregulate MACC1. *Frontiers in Genetics*.

[10] Ye C., Shen Z., Wang B. (2016). A novel long non-coding RNA lnc-GNAT1-1 is low expressed in colorectal cancer and acts as a tumor suppressor through regulating RKIP-NF-kappaB-Snail circuit. *Journal of Experimental & Clinical Cancer Research : CR*.

[11] Zhang N., Hu X., He S. (2019). LncRNA MSC-AS1 promotes osteogenic differentiation and alleviates osteoporosis through sponging microRNA-140-5p to upregulate BMP2. *Biochemical and Biophysical Research Communications*.

[12] Hu Z., Li L., Cheng P. (2020). lncRNA MSC‐AS1 activates Wnt/*β*‐catenin signaling pathway to modulate cell proliferation and migration in kidney renal clear cell carcinoma via miR‐3924/WNT5A. *Journal of Cellular Biochemistry*.

[13] Bian Z., Jin L., Zhang J. (2016). LncRNA-UCA1 enhances cell proliferation and 5-fluorouracil resistance in colorectal cancer by inhibiting miR-204-5p. *Scientific Reports*.

[14] Zhu S., Chen C. Y., Hao Y. (2021). LncRNA KCNQ1OT1 acts as miR-216b-5p sponge to promote colorectal cancer progression via up-regulating ZNF146. *Journal of Molecular Histology*.

[15] Zhang Y. H., Fu J., Zhang Z. J., Ge C. C., Yi Y. (2016). LncRNA-LINC00152 down-regulated by miR-376c-3p restricts viability and promotes apoptosis of colorectal cancer cells. *American Journal of Translational Research*.

[16] Sun S., Liu F., Xian S., Cai D. (2020). miR-325-3p overexpression inhibits proliferation and metastasis of bladder cancer cells by regulating MT3. *Medical Science Monitor : International Medical Journal of Experimental and Clinical Research*.

[17] Alaiyan B., Ilyayev N., Stojadinovic A. (2013). Differential expression of colon cancer associated transcript1 (CCAT1) along the colonic adenoma-carcinoma sequence. *BMC Cancer*.

[18] Sun W., Nie W., Wang Z., Zhang H., Li Y., Fang X. (2020). Lnc HAGLR promotes colon cancer progression through sponging miR‐185‐5p and activating CDK4 and CDK6 in vitro and in vivo. *OncoTargets and Therapy*.

[19] Gu J.-X., Zhang X., Miao R.-C. (2019). Six-long non-coding RNA signature predicts recurrence-free survival in hepatocellular carcinoma. *World Journal of Gastroenterology*.

[20] Cao C., Zhong Q., Lu L. (2020). Long noncoding RNA MSC‐AS1 promotes hepatocellular carcinoma oncogenesis via inducing the expression of phosphoglycerate kinase 1. *Cancer Medicine*.

[21] Zhang L., Zhao G., Ji S., Yuan Q., Zhou H. (2020). Downregulated long non-coding RNA MSC-AS1 inhibits osteosarcoma progression and increases sensitivity to cisplatin by binding to MicroRNA-142. *Medical Science Monitor : International Medical Journal of Experimental and Clinical Research*.

[22] Li C., Feng S., Chen L. (2021). MSC-AS1 knockdown inhibits cell growth and temozolomide resistance by regulating miR-373-3p/CPEB4 axis in glioma through PI3K/Akt pathway. *Molecular and Cellular Biochemistry*.

[23] Zhang L., Chen H., Song Y. (2020). MiR-325 promotes oxaliplatin-induced cytotoxicity against colorectal cancer through the HSPA12B/PI3K/AKT/Bcl-2 pathway. *Digestive diseases and sciences*.

[24] Chen M., Meng Q., Qin Y. (2016). TRIM14 inhibits cGAS degradation mediated by selective autophagy receptor p62 to promote innate immune responses. *Molecular Cell*.

[25] Tan Z., Song L., Wu W. (2018). TRIM14 promotes chemoresistance in gliomas by activating Wnt/*β*-catenin signaling via stabilizing Dvl2. *Oncogene*.

[26] Hai J., Zhu C. Q., Wang T., Organ S. L., Shepherd F. A., Tsao M. S. (2017). TRIM14 is a putative tumor suppressor and regulator of innate immune response in non-small cell lung cancer. *Scientific Reports*.

[27] Jin Z., Li H., Hong X. (2018). TRIM14 promotes colorectal cancer cell migration and invasion through the SPHK1/STAT3 pathway. *Cancer Cell International*.

[28] Shen W., Jin Z., Tong X. (2019). TRIM14 promotes cell proliferation and inhibits apoptosis by suppressing PTEN in colorectal cancer. *Cancer Management and Research*.

